# Challenges in the Diagnosis and Management of Spinal Tuberculosis: Case Series

**DOI:** 10.7759/cureus.3855

**Published:** 2019-01-08

**Authors:** Anudariya Dean, Stephanie Zyck, Gentian Toshkezi, Michael Galgano, Satya Marawar

**Affiliations:** 1 Neurosurgery, State University of New York Upstate Medical University, Syracuse, USA; 2 Neurosurgery, Thomas Jefferson University Hospitals, Philadelphia, USA; 3 Orthopaedics, Syracuse Veterans Affairs Hospital, Syracuse, USA

**Keywords:** spinal tuberculosis, pott disease, tuberculosis

## Abstract

Though uncommon in developed countries, spinal tuberculosis must still be considered in patients with a suspicious clinical history, to avoid delays in treatment. This case series highlights the special considerations that need to be taken into account while tackling the diagnostic and therapeutic challenges associated with this disease.

We present two interesting cases of spinal tuberculosis. The first case was a 26-year-old female who presented with chronic back pain and an initial misdiagnosis of ankylosing spondylitis. The second case was a 26-year-old male with new lower extremity weakness, numbness, and urinary retention.

Both cases had clear indications for surgery, however, the first case was treated with medical management upon patient request. The patient was managed non-surgically and improved clinically though she may need surgery in the future. The second case was treated with emergency surgery and the patient regained full neurologic function at follow-up.

These cases demonstrate the importance of considering spinal tuberculosis in the differential diagnosis of high-risk patients as well as individualizing treatment strategies for each patient.

## Introduction

Though rare in developed countries, *Mycobacterium tuberculosis *remains a major global problem and is one of the top 10 causes of death worldwide [1]. Spinal tuberculosis, also known as Pott's disease, is a significant source of morbidity for patients with extrapulmonary disease. Back pain is the most common and earliest presenting symptom, followed by other findings, such as progressive neurologic deficits and spinal deformity. However, some patients may lack clinical symptoms even in the presence of active spinal disease. Constitutional symptoms present in only 20%-30% of cases [2]. Lack of suspicion in non-endemic areas, paired with an insidious presentation of symptoms can occasionally lead to delays in diagnosis and treatment. 

In developing countries, spinal tuberculosis is usually diagnosed with clinical features and plain radiographs. Plain radiographs may show radiolucent lesions, joint space narrowing, anterior wedging of vertebral bodies, kyphosis, and calcified abscesses of soft tissue. However, the disease must be significantly progressed before these findings are visible. In developed countries, magnetic resonance imaging (MRI) and computed tomography (CT) are utilized more frequently. Computed tomography is the most sensitive study for characterizing bony involvement at an early stage as well as pathognomonic calcifications within tuberculous abscesses. Soft tissue and spinal canal involvement are best evaluated with an MRI [2]. Earlier diagnosis and increased sensitivity for smaller lesions are other advantages of MRI. Once identified on imaging, the confirmation of diagnosis then requires a positive culture from an abscess or bone biopsy [2].

The differential diagnosis of tuberculous spondylitis includes pyogenic spondylitis and malignancy. End plate disruption, sub-ligamentous spread, and intraosseous and paravertebral abscesses are characteristic findings of spinal tuberculosis. Hyperintensity of the disc space is often seen on T2-weighted MRI in advanced stages though a narrowing of the disc space typically doesn't occur until a significant degeneration of neighboring vertebral bodies has occurred. While paraspinal abscesses are frequently present with other types of infection, tuberculosis abscess walls have several differentiating features. With Pott's disease, abscess walls tend to be thin, smooth, and without a surrounding inflammatory response. In contrast, pyogenic spondylitis tends to have thick abscess walls with irregular contrast enhancement. Another differentiating feature is more frequent disc involvement with pyogenic spondylitis. Spinal tumors can also have vertebral bone destruction and local masses on MRI, though intervertebral disk involvement is rare [2]. Thus, differentiation between spinal tumors and tuberculosis is challenging in the early stages of tuberculous spondylitis where an intervertebral disk has not been involved yet. In such cases, a thorough history and details of the clinical presentation are important in making an accurate diagnosis. A needle biopsy also helps to differentiate between pathologies; however, in the presence of compressive pathology and significant neurologic decline, this may not be possible before open surgery is required.

We present two cases that demonstrate the diagnostic and management challenges that we face in treating spinal tuberculosis today.

## Case presentation

Case 1

A 26-year-old female healthcare worker who had immigrated from India several years prior presented to her primary care physician with an acute onset of severe back pain after a trip home to India. The pain radiated down both legs, was worse at night and with activity, and was relieved with naproxen and oral methylprednisone. Past medical, surgical, and social history were otherwise unremarkable. A mechanical etiology was suspected and the patient was managed conservatively with physical therapy. Over the following weeks, she developed urinary frequency, constipation, and dysmenorrhea. She was initially diagnosed with pelvic floor dysfunction. Her back pain continued to progressively worsen over the following months, and she was started on prednisone. She did report unintentional weight loss but denied other constitutional symptoms.

**Figure 1:**
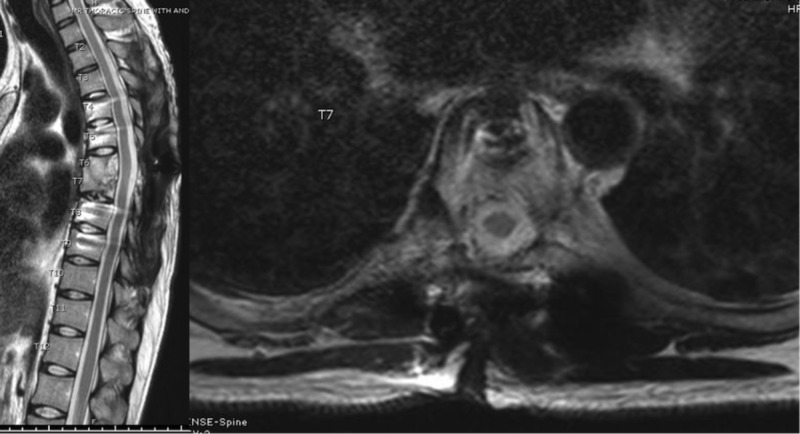
MRI thoracic spine Magnetic resonance imaging (MRI) of the thoracic spine sagittal (right) and axial (left) T2 sequence showing resolution of the epidural collection and stable signal change in the superior aspect of the T7 vertebral body without any evidence of disease progression.

Her physical exam remained normal, except for right sacroiliac joint and lumbar spinal tenderness. The straight leg raising test was also positive bilaterally. Rheumatologic markers were unremarkable though her white cell count was elevated at 12.9. An infectious disease specialist was consulted and felt that infectious disease was unlikely in the setting of a normal C-reactive protein (CRP) and erythrocyte sedimentation rate (ESR). An MRI of the pelvis and lumbar spine demonstrated bone marrow edema in the inferior aspect of the right sacroiliac joint, thought to be consistent with an early stage of sacroiliitis. A Schmorl's node at L4 was also reported (Figure 1). Based on the clinical and MRI findings, the patient was diagnosed with early-stage ankylosing spondylitis. The rheumatology service was consulted, and she was treated with hydroxychloroquine and prednisone. Hydroxychloroquine was stopped after two weeks due to the development of myalgia and visual floaters. Immunosuppressive therapy was proposed as an alternative, so a QuantiFERON-TB Gold test^©^ (Qiagen, Hilden, Germany) was ordered. This resulted as positive. Interestingly, a purified protein derived (PPD) skin test one year prior had been negative and a chest X-ray was also within normal limits.

Although the spinal lesion seen did not have the characteristic features seen in spinal tuberculosis, a needle biopsy was performed. This revealed necrotizing granulomas with rare acid-fast bacilli. The sputum analysis was then found to be positive for *Mycobacterium tuberculosis*. The patient was started on a four-drug regimen of isoniazid, rifampin, ethambutol, and pyrazinamide. The sputum culture was negative twice after two weeks of treatment.

**Figure 2:**
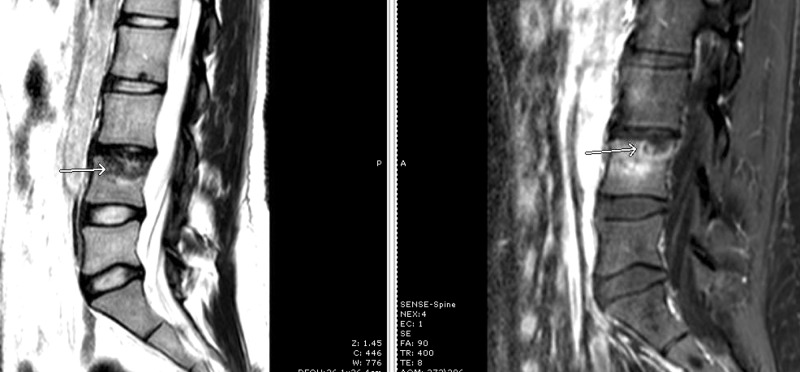
Initial MRI lumbar spine Magnetic resonance imaging (MRI) sagittal view with T2-weighted (left) and contrast-enhanced T1-weighted (right) sequences showing signal abnormality in the superior endplate of the L4 vertebral body, initially interpreted as a Schmorl's node (arrows).

**Figure 3:**
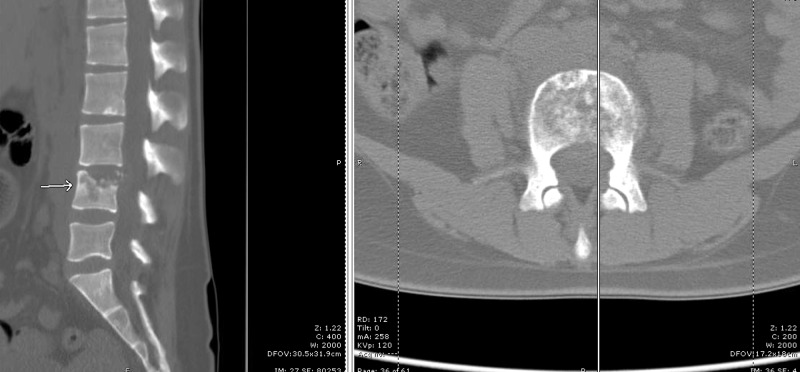
CT lumbar spine Sagittal (left) and axial (right) views of computed tomography (CT) of the lumbar spine showing lytic changes at the L4 vertebral body (arrow) and mild narrowing of the L3-4 disc space

Despite medical management, she was admitted to the hospital a year after the onset of her symptoms with fever, severe nausea, and emesis. Repeat CT of the lumbar spine without contrast showed a lytic lesion in the L4 vertebral body with a partial compression fracture and mild retropulsion of bone into the ventral spinal canal (Figure 2). A repeat lumbar MRI showed edema and enhancement of the L4 vertebral body with erosive changes and subligamentous spread consistent with tuberculous discitis and osteomyelitis. There was an epidural collection at the L3-4 level, with sub-articular zone involvement (Figure 3). The left neural foramen and exiting nerve root were also involved. At this point in time, the neurosurgery service was consulted. Due to the small size of the epidural component, a stable neurologic exam without any focal deficits, patient preference, and aggressive medical management was recommended.

**Figure 4:**
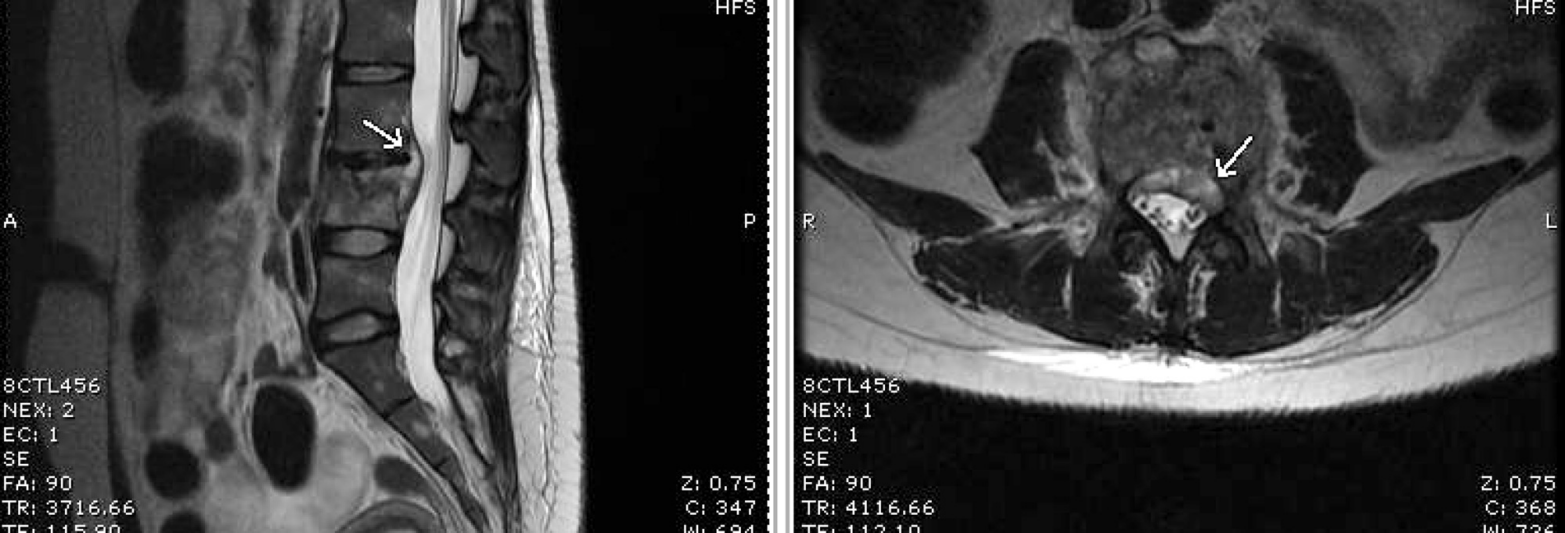
Follow-up lumbar MRI Magnetic resonance imaging (MRI) study with sagittal (left) and axial (right) T2-weighted views showing signal change within the L4 vertebral body with subligamentous spread (arrow on sagittal view) and an epidural component and sub-articular involvement (arrow on axial view)

However, the patient continued to have a persistent fever above 38°C, worsening radicular pain, and declining functional status. The follow-up MRI revealed an expanding epidural abscess and eroded lumbar vertebrae with progressive kyphosis as compared with two months prior (Figure 4). Her spine was stabilized with a thoracolumbosacral orthosis (TLSO) brace to minimize the risk of neurological decline. Posterior lumbar decompression with an evacuation of the abscess, L4 corpectomy, anterior column reconstruction, and instrumented fusion from L2-S1 were strongly recommended. However, the patient refused surgical treatment and requested to continue with conservative management.

**Figure 5:**
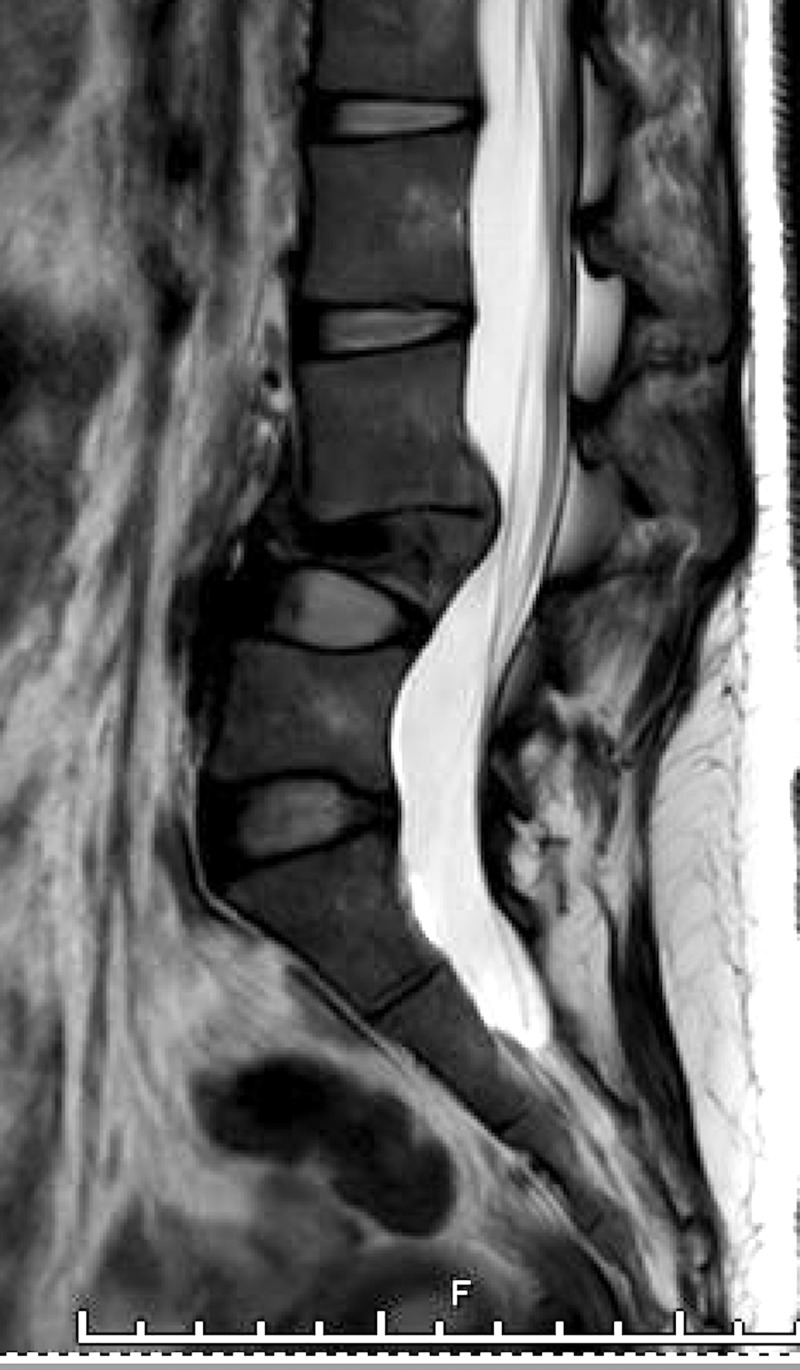
Lumbar MRI with new deformity Sagittal T2-weighted MRI showing increased compression and anterior wedging of L4 vertebral body with subsequent kyphosis.

A second opinion was then sought from an outside hospital. A two-month course of intravenous amikacin was added to her antimicrobial regimen. Despite the physical deformity and our continued recommendation for surgery, the patient has continued to prefer non-surgical management. Her most recent MRI performed 18 months after the diagnosis of spinal tuberculosis and initiation of anti-tuberculosis therapy showed decreased evidence of osteomyelitis and resolution of the psoas collection (Figure 5), A plain radiograph was also performed, which showed stable loss of vertebral body height and stable grade 1 spondylolisthesis at L3-4, without any increase in angular deformity or motion seen in the flexion-extension views (Figure 5). The patient has continued to request non-surgical management and has since requested not to undergo any additional follow-up imaging. At the most recent clinical follow-up at 22 months after initial diagnosis, she is neurologically intact and ambulatory with minimal back pain.

Case 2

A 26-year-old male prisoner presented with a sudden onset of bilateral loss of motor and sensory function below the waist and urinary retention for two days. He reported an eight-month history of chronic mid-thoracic pain and a three-month history of progressive sensory loss over his feet. He denied recent trauma or illness. His PPD five months’ prior was negative. He denied smoking or a history of intravenous drug use. The physical exam was significant for saddle anesthesia, absent strength in the bilateral lower extremities, diminished sensation to pinprick below the T4 level, and hyperreflexia in both lower extremities. Basic labs, human immunodeficiency virus (HIV) testing, and a hepatitis panel were normal.

**Figure 6:**
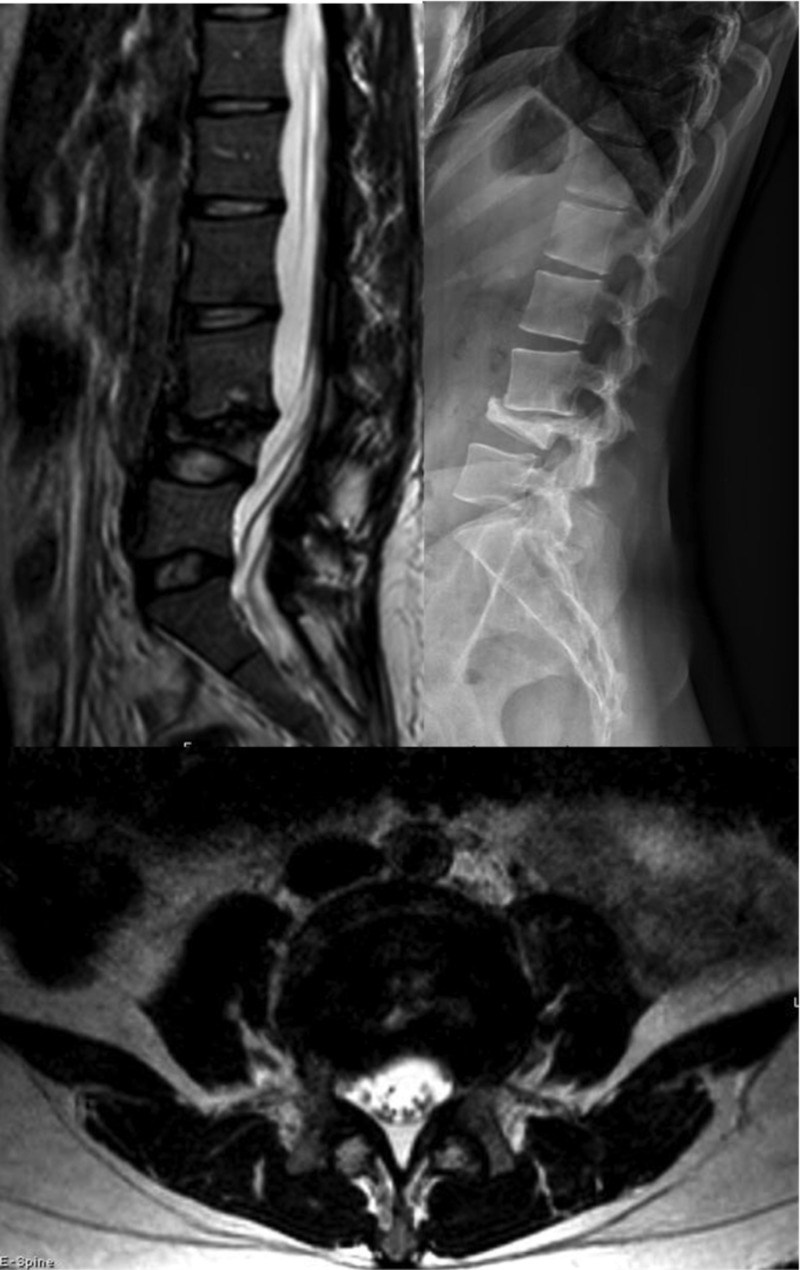
MRI thoracic spine and thoracic spine radiograph Magnetic resonance imaging (MRI) of the thoracic spine T2 sagittal (top left) and axial (bottom) views showed decreased evidence of osteomyelitis and resolution of the psoas collection. A plain radiograph flexion-extension (top right) showed stable loss of vertebral body height and stable grade 1 spondylolisthesis at L3-L4, without any increase in angular deformity or motion.

A thoracic MRI with and without contrast demonstrated a peripherally enhancing collection centered within the T6-T7 disc space. Endplate erosion, mild retrolisthesis, paraspinal extension, and epidural involvement causing severe cord compression were seen. Subligamentous spread beneath the anterior longitudinal ligament from T4 through T8 was also seen (Figure 6). The findings were consistent with discitis and osteomyelitis. Given the subligamentous spread, tuberculosis was considered in the differential.

CT of the thoracic spine re-demonstrated erosion of the T6 and T7 vertebral bodies, pedicles, and adjacent ribs. A heterogeneous, predominantly low-density collection extending into the paraspinal region, subligamentous space, and epidural space was also visible on the CT scan. In addition, patchy parenchymal opacity was present in the apex of the right lung. A thoracic CT was then performed, which showed diffuse pleural thickening on the right, with more focal pleural plaques and multiple pulmonary nodules.

**Figure 7:**
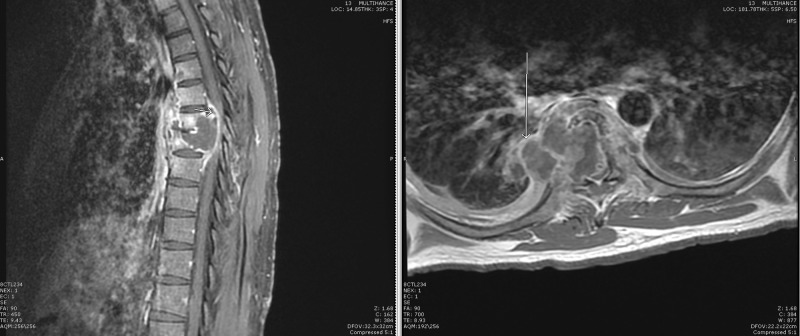
MRI thoracic spine Sagittal (left) and axial (right) views of contrast-enhanced T1-weighted magnetic resonance imaging (MRI) showing abnormal signal with loss of vertebral body height and mild retrolisthesis at T6 and T7. There is also a peripherally enhancing collection within the T6-7 disc space extending into the paraspinal soft tissues and epidural space, causing cord compression. Subligamentous spread is seen anteriorly from the T4 through T8 levels.

The patient underwent emergent T6-7 hemilaminectomy with partial costovertebrectomy and T4-9 posterior spinal fusion (Figure 7). Fluorochrome stain from intraoperative cultures revealed acid-fast bacilli. Anaerobic and fungal cultures did not grow any organisms. A QuantiFERON-TB Gold test^©^ (Qiagen, Hilden, Germany) was positive for *Mycobacterium tuberculosis*.

**Figure 8:**
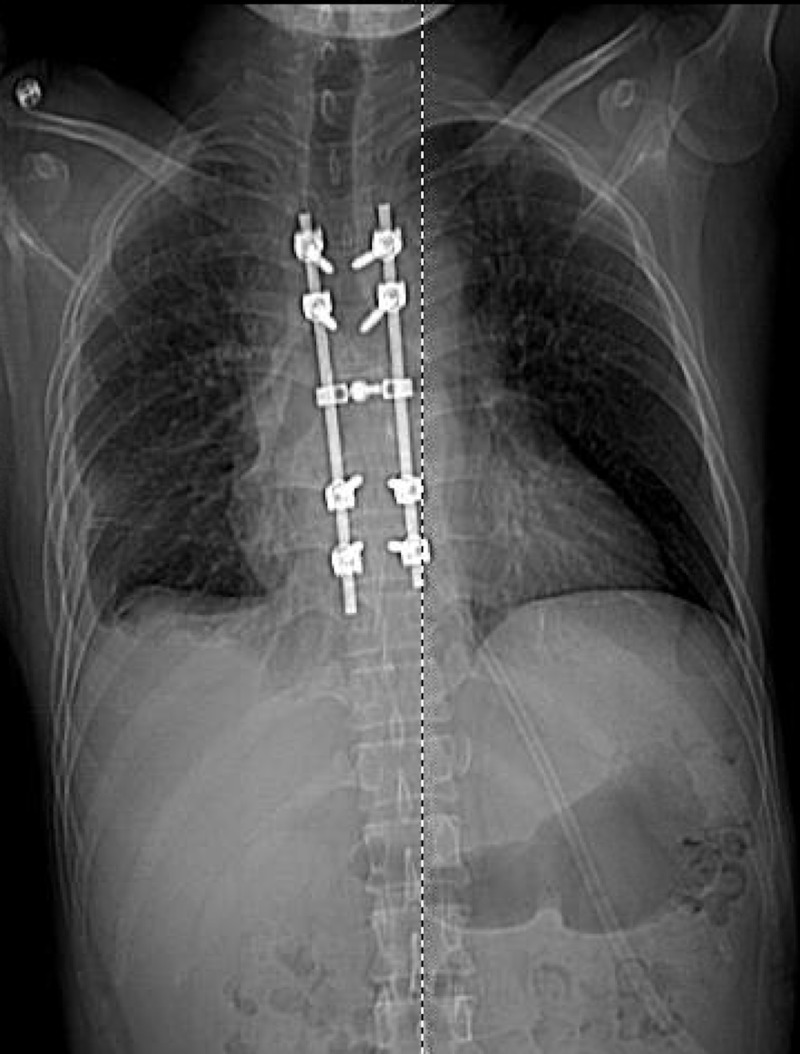
Postoperative radiograph Postoperative radiograph, anteroposterior view, shows thoracic hardware from T4-9 levels.

On the first post-operative day, lower extremity motor examination had improved to a 4+/5 weakness of the iliopsoas and quadriceps bilaterally. Distal lower extremity musculature, including hamstrings, anterior tibialis, and gastrocnemius, were graded at 1/5. The patient was started on rifampin, isoniazid, pyrazinamide, and ethambutol. The patient was discharged to acute inpatient rehabilitation on the ninth postoperative day. At discharge, he had full motor strength in the lower extremities with the exception of 3/5 bilateral extensor hallucis longus strength. He continued to have sensory deficits in the lower extremities bilaterally. A month and a half following discharge, the patient was readmitted to the hospital due to a wound infection, which was debrided and treated with antibiotics. At the most recent follow-up one-year postoperatively, the patient was neurologically intact. Follow-up imaging showed the stable appearance of the hardware, resolution of the epidural collection, and stable signal change in the superior aspect of the T7 vertebral body without any evidence of disease progression (Figure 8).

## Discussion

While spinal tuberculosis is not commonly seen in developed countries, it is endemic in many developing nations. As the air travel for tourism and business has become common in and out of the endemic areas of the USA, there is a need for knowledge of the clinical presentation and management of various presentations of tuberculosis. As demonstrated in our two cases, spinal tuberculosis clinically and radiographically can mimic other pathologies. This diagnostic challenge often leads to a delay in appropriate treatment. In our first case, the patient was initially misdiagnosed as having ankylosing spondylitis. She was started on a course of prednisone and hydroxychloroquine. Subsequently, her disease progressed. Once a needle biopsy was performed with cultures growing *Mycobacterium tuberculosis*, the correct diagnosis was reached and an appropriate antibiotic regimen was started. This example demonstrates the importance of performing a needle biopsy in cases of diagnostic uncertainty, as well as keeping Pott’s disease in the differential diagnosis even when not in a tuberculosis-endemic area. Another interesting point from our cases was the negative PPD test in both of our patients with Pott's disease.

Both patients in our series did have a clinical history and radiographic findings that offered valuable insight to distinguish a tuberculosis infection from other pathologies. The subligamentous spread, thin and smooth paraspinal abscess walls, and intervertebral disc abscess walls seen in our cases were consistent with spinal tuberculosis, as was calcification seen on CT imaging. Both of our patients also had a history suggestive of exposure to tuberculosis. Therefore, histories with a high risk of exposure to infection such as immigration, incarceration, immunocompromise, or employment in healthcare should all lead to a heightened suspicion for an infectious etiology of spinal disease.

Spinal tuberculosis can be managed either medically or surgically. Optimal prognosis and recovery are associated with the initiation of treatment at the earliest stage possible [2]. As a systemic infectious disease, medical management includes an aggressive, anti-tubercular, anti-microbial regimen in addition to physical therapy and immobilization. Some studies have shown that routine surgical treatment doesn’t provide a long-term benefit as compared with medical management. One randomized control trial by the Medical Research Council Working Party compared combined surgical and medical intervention to medical intervention alone. It studied 130 patients who were treated with isoniazid and amino-salicylate sodium (sodium PAS) with or without operative debridement within two months of the start of medical therapy [3]. Another randomized controlled trial evaluated 201 patients who were treated with six months of isoniazid plus rifampicin with or without radical anterior resection with bone grafting [4]. The results of both studies at the five-year assessment revealed no added benefit of combined therapy over medical therapy alone. However, both of these studies excluded patients with advanced diseases. Surgical management is generally indicated for cases requiring tissue samples to establish a diagnosis, disease refractory to medical treatment, neurologic compromise, spinal instability and deformity [5]. 

Several studies have been performed to try to more precisely define surgical candidacy. A prospective observational study by Rajasekaran et al., evaluated 61 children with Pott's disease who were treated with medical therapy during a 15-year period [6]. It was found that unlike in adults, spinal deformity in children during the active phase of the disease progresses even after the completion of treatment, especially during growth. The final spinal deformity can be predicted by a scoring system based on the spinal radiographic signs. One possible conclusion of this study is that children with a predicted final spinal deformity with kyphosis of 30-60 degrees are early surgical candidates [6]. In adults, a Cochrane analysis of two trials revealed no statistically significant difference in outcomes, including the degree of deformity, neurologic status, bony fusion, resolution of spinal tuberculosis, mortality, or functionality [5]. However, both studies excluded patients with paraplegia, disease refractory to medical treatment, or significant extra-spinal disease [5]. Thus, the recommendation loses applicability to the population of patients who would not meet those criteria. 

Furthermore, the cervical or thoracic spine may tolerate a lower degree of compression than the lumbar spine due to cord involvement and the risk of venous thrombosis. It is our opinion that the involvement of the cervical or thoracic spine should significantly lower the threshold for urgent aggressive surgical intervention even in the presence of a stable neurologic exam due to the high risk of venous thrombosis of the spinal cord and subsequent neurologic decline.

Multiple classifications of spinal tuberculosis have been developed to try to give clear indications for medical versus surgical management. Kumar, in 1985, presented his classification of four points based on the location and stages of the posterior spinal tuberculosis [7]. This classification is considered to have limited utility since posterior spinal tuberculosis is not a common form of Pott’s disease. Mehta and Bhojraj attempted to improve the classification in 2001. Their classification was based on MRI findings, classifying patients again into four groups. Group A included patients without spinal instability or kyphosis that required only anterior debridement. Group B included patients with spinal instability and kyphosis who required posterior instrumentation. Group C included patients with extended anterior lesions. They were reported to be best managed through posterior decompression and instrumentation with the transpedicular approach to the anterior vertebral column due to the high risk of the transthoracic approach. Lastly, group D patients presented with only posterior tuberculosis that required posterior decompression [8].

Oguz et al. introduced another classification, with three types based on retrospective analysis of 76 cases. In Type I, the disease involved a one-level disc and soft tissue without abscess, collapse, or neurologic deficit. In Type I-A, lesions were limited to vertebra only so needle biopsy confirmed the diagnosis and medical management was then utilized from that point forward. Type I-B described cases in which there was an abscess that exceeded the vertebra. These cases thus required debridement through either an anterior, posterior, or endoscopic approach. Type II described cases with one- or two-level disc involvement with abscess formation and mild kyphosis. They were corrected with an anterior surgical approach. Patients with Type III disease had one- or two-level disc degeneration, abscess, and spinal instability that required decompression and instrumentation through the anterior, posterior, or combined approaches [9].

Our first patient had progressive kyphosis, leading to impaired function, and our second patient had a new onset of lower extremity weakness. Both patients in our series could have benefitted from surgical treatment, though only our second patient underwent surgery due to personal preferences. In that case, that patient had full capacity and an adequate understanding of her disease process and strongly wished to avoid surgical intervention. Fortunately, at the most recent follow-up, she has clinically stabilized with conservative management.

## Conclusions

While spinal tuberculosis is a rare condition in developed countries, it must still be considered in the differential diagnosis for patients presenting with back pain, systemic symptoms, or new neurologic deficits, especially when clinical presentation and imaging findings are suspicious. Needle biopsy is useful in cases that are diagnostically uncertain. Surgical decompression, biopsy, and stabilization are indicated for patients with progressive neurologic deficits, refractory symptoms, or progressive deformity. We used our case presentations to highlight the importance of maintaining spinal tuberculosis in the differential diagnosis and discussed the factors that must be considered when developing a treatment plan.
